# The Logistics and Coordination of Respiratory Syncytial Virus Immunoprophylaxis Use Among US Pediatric Specialists

**DOI:** 10.1177/0009922815621343

**Published:** 2016-01-08

**Authors:** Pierre C. Wong, Prabhu S. Parimi, Joseph B. Domachowske, Deborah M. Friedman, Michael G. Marcus, Daniel F. Garcia, William V. La Via, Iqra A. Syed, Shelagh M. Szabo, Kimmie K. McLaurin, Veena R. Kumar

**Affiliations:** 1 Children’s Hospital Los Angeles, Los Angeles, CA, USA; 2 University of Kansas Hospital, Kansas City, KS, USA; 3 Upstate Medical University, Syracuse, NY, USA; 4 New York Medical College, Valhalla, NY, USA; 5 Maimonides Medical Center, New York, NY, USA; 6 Arnold Palmer Hospital, Orlando, FL, USA; 7 AstraZeneca, Gaithersburg, MD, USA; 8 ICON, Vancouver, BC, Canada

**Keywords:** RSV, palivizumab, neonatologist, pediatrician, pulmonologist, cardiologist

## Abstract

This study was conducted to survey US pediatric specialists about administration of respiratory syncytial virus (RSV) immunoprophylaxis, communication patterns among physicians and parents, and barriers to access. Separate surveys were sent to neonatologists, pediatricians, pediatric pulmonologists, and pediatric cardiologists. Most physicians (≥93.5%) routinely recommended immunoprophylaxis to high-risk children. Most respondents (≥71.8%) reported that >50.0% of eligible infants and young children received each monthly dose throughout the RSV season, with the first dose most commonly administered before discharge from the birth hospitalization. To ensure receipt of subsequent doses, specialists frequently scheduled a follow-up visit at the end of the current appointment. All specialists reported insurance denials as the biggest obstacle to the administration of immunoprophylaxis to high-risk children. These findings may be used to improve adherence to immunoprophylaxis by enhancing education and physician-parent communications about severe RSV disease prevention, and by reducing known barriers to use of this preventive therapy.

## Introduction

Respiratory syncytial virus (RSV) is the leading cause of infant pneumonia and bronchiolitis,^1^ and is the leading cause of hospitalizations among infants and young children in the United States.^2,3^ In certain high-risk populations—preterm infants ≤35 weeks’ gestational age (wGA) who are ≤6 months of age and children ≤24 months of age with bronchopulmonary dysplasia (BPD)/chronic lung disease of prematurity (CLDP) or hemodynamically significant congenital heart disease (HS-CHD)—the monoclonal antibody palivizumab has been shown to significantly reduce the risk of hospitalizations attributable to severe RSV disease.^3,4^ The American Academy of Pediatrics (AAP) publishes regularly updated recommendations for the use of RSV immunoprophylaxis to help reduce RSV-related hospitalizations in high-risk children. The 2012 AAP guidance provided recommendations for RSV immunoprophylaxis use among preterm infants ≤34 wGA and children with BPD/CLDP or HS-CHD.^5^ In July 2014, the guidance was updated, with the most significant changes being that RSV immunoprophylaxis use among preterm infants without BPD/CLDP was limited to those ≤28 wGA, and for children with HS-CHD its use was no longer routinely recommended in the second year of life.^6^

Research on how palivizumab is used in clinical practice has been limited. In particular, how often RSV immunoprophylaxis is recommended by physicians, the reasons physicians do not recommend immunoprophylaxis in certain circumstances, and the communication patterns between and among physicians and with their patients’ parents regarding immunoprophylaxis administration have not been well studied. Moreover, even though palivizumab use is recommended by the AAP, literature on how these recommendations translate into real-world clinical practice is limited.

## Objectives

The objectives of the survey were to understand (*a*) the current clinical practice for the provision of RSV immunoprophylaxis by neonatologists, pediatricians, pediatric pulmonologists, and pediatric cardiologists in the United States; (*b*) the strategies utilized by these specialists to achieve compliance and their perceived barriers to access; and (*c*) how information regarding the need for RSV immunoprophylaxis is communicated between pediatric specialists and primary care providers.

## Methods

### Study Design

In early 2014, before the release of the updated 2014 AAP guidance, specialty-specific cross-sectional surveys were developed and administered online to neonatologists and pediatricians (online Appendix I; supplementary material can be found at http://clp.sagepub.com/supplemental), pediatric pulmonologists (online Appendix II), and pediatric cardiologists (online Appendix III) who practiced in the United States. This study was conducted in accordance with the Declaration of Helsinki and was consistent with International Conference on Harmonization Good Clinical Practice, Good Epidemiology Practices, and applicable regulatory requirements. Institutional review board approval was not required because this study involved the administration of surveys without disclosure of protected health information.

### Target Population and Sample

All physicians who self-identified as neonatologists, pediatric pulmonologists, or pediatric cardiologists practicing in the United States and who were part of the American Medical Association (AMA) database were invited via email to participate in the online survey. The sample of pediatricians who received the survey was randomly selected because of their larger numbers when compared with the subspecialists. Physicians who recommended RSV immunoprophylaxis for high-risk children were eligible to complete the survey. Participants who did not complete the survey after receiving the initial email invitations were sent subsequent email reminders. All respondents had the option to accept an honorarium of $75.

For each group of neonatologists, pediatricians, pediatric pulmonologists, and pediatric cardiologists, the target sample size was up to 200 physicians, consistent with a recent survey of physicians assessing patient compliance with RSV immunoprophylaxis.^7^ Obtaining responses from a large representative sample of physicians from each specialty, from a broad variety of practice types, and with various years of clinical experience and patient populations were the main considerations for this sample size.

### Outcomes and Analytic Methods

Data were collected using structured and open-ended questions. The survey for neonatologists and pediatricians focused on preterm infants without CLDP or congenital heart disease (CHD), whereas the surveys for pediatric pulmonologists and pediatric cardiologists were specific to children with CLDP or HS-CHD, respectively. Outcomes from the survey pertaining to objective (*a*)—the current clinical practice of RSV immunoprophylaxis use by the respondents—have been previously reported in a separate publication.^8^ The survey outcomes presented herein pertain to the questions that focused on objective (*b*)—that is, where eligible children receive their first and subsequent doses of RSV immunoprophylaxis, what strategies are used by physicians to ensure compliance, and what their perceived barriers are to RSV immunoprophylaxis access, and objective (*c*)—that is, how information on the need for RSV immunoprophylaxis is communicated between and among physicians and with parents.

Survey responses were analyzed separately according to specialty type. The responses from physicians indicating who they considered primarily responsible for prescribing RSV immunoprophylaxis, according to setting, were tabulated. A similar process was undertaken to describe the biggest obstacles to, and methods to facilitate provision of, RSV immunoprophylaxis. Responses to the survey were also used to explore how different physician specialists communicated with each other and with parents.

## Results

Among the neonatologists (n = 4544), pediatric pulmonologists (n = 1010), pediatric cardiologists (n = 2382), and a random sample of pediatricians (n = 7311) in the AMA database who were contacted, 203 neonatologists, 138 pediatricians, 58 pediatric pulmonologists, and 156 pediatric cardiologists completed the survey. Their demographic and practice characteristics are presented in Table 1. A schematic of the number of physicians who were invited, who initiated, and who completed the survey is provided in online Appendix IV.

**Table 1.** Respondents and Practice Characteristics.

Characteristic, n (%)	Neonatologists^a^ (n = 203)	Pediatricians (n = 138)	Pediatric Pulmonologists (n = 58)	Pediatric Cardiologists (n = 156)
Male	128 (63.1)	62 (44.9)	37 (63.8)	105 (67.3)
US location^b^				
Northeast	63 (31.0)	37 (26.8)	19 (32.8)	41 (26.3)
North Central	52 (25.6)	27 (19.6)	12 (20.7)	31 (19.9)
South	58 (28.6)	43 (31.2)	17 (29.3)	47 (30.1)
West	30 (14.8)	31 (22.5)	10 (17.2)	36 (23.1)
US possessions	0	0	0	1 (0.6)
Year of graduation from residency or fellowship				
Before 1985	22 (10.8)	10 (7.2)	3 (5.2)	12 (7.7)
1985-1994	38 (18.7)	35 (25.4)	9 (15.5)	29 (18.6)
1995-2004	44 (21.7)	42 (30.4)	19 (32.8)	40 (25.6)
2005-2014	88 (43.3)	44 (31.9)	27 (46.6)	75 (48.1)
2015-2016	11 (5.4)	7 (5.1)	—	—
Practice type				
Medical school–based teaching hospital	97 (47.8)	25 (18.1)	41 (70.7)	109 (69.9)
Non–medical school teaching hospital	41 (20.2)	8 (5.8)	6 (10.3)	17 (10.9)
Nonteaching community hospital	28 (13.8)	7 (5.1)	0	2 (1.3)
Large group practice (≥5 pediatric specialists)	28 (13.8)	49 (35.5)	3 (5.2)	17 (10.9)
Small group practice (<5 pediatric specialists)	9 (4.4)	33 (23.9)	3 (5.2)	8 (5.1)
Individual practice	0	16 (11.9)	5 (8.6)	3 (1.9)
Primary area^c^				
NICU	201 (99.0)	13 (9.4)	—	—
Inpatient	45 (22.2)	45 (32.6)	40 (69.0)	43 (27.6)
Outpatient	10 (4.9)	122 (88.4)	56 (96.6)	112 (71.7)
Catheterization lab	—	—	—	27 (17.3)
Imaging	—	—	—	47 (30.1)
Intensive care unit	—	—	15 (25.9)	31 (19.9)
Research	—	—	24 (41.4)	12 (7.7)
Community type				
Inner city only	21 (19.6)^d^	18 (13.0)	4 (6.9)	14 (9.0)
Urban (non–inner city) only	30 (28.0)^d^	23 (16.7)	14 (24.1)	54 (34.6)
Suburban only	25 (23.4)^d^	55 (39.9)	12 (20.7)	27 (17.3)
Rural only	5 (4.7)^d^	11 (8.0)	1 (1.7)	3 (1.9)
Multiple	26 (24.3)^d^	31 (22.5)	27 (46.5)	58 (37.2)
Number of preterm infants (≤12 months of age) born at ≤35 wGA without CLDP or HS-CHD cared for in the past 12 months				
1-30	38 (18.7)	109 (79.0)	—	—
31-60	70 (34.5)	16 (11.6)	—	—
61-250	95 (46.8)	13 (9.4)	—	—
Children with CLDP or HS-CHD^e^ in practice				
Cared for in the past 12 months				
1-50	—	—	41 (70.7)	96 (61.5)
51-100	—	—	12 (20.7)	31 (19.9)
101-500	—	—	5 (8.6)	24 (15.4)
501-1000	—	—	0	2 (1.3)
1001-5000	—	—	0	3 (1.9)
Cared for and received RSV immunoprophylaxis in the past 12 months (%)				
<20	—	—	2 (3.4)	21 (13.5)
20-39	—	—	9 (15.5)	32 (20.5)
40-59	—	—	11 (19.0)	18 (11.5)
60-79	—	—	10 (17.2)	28 (17.9)
80-100	—	—	26 (44.8)	57 (36.5)

Abbreviations: CLDP, chronic lung disease of prematurity; HS-CHD, hemodynamically significant congenital heart disease; NA, not applicable; NICU, neonatal intensive care unit; RSV, respiratory syncytial virus; wGA, weeks’ gestational age.

a Among neonatologists, the highest levels of nursery service were as follows: Level 1 (ie, normal care nursery), n = 1 (0.5%); Level 2 (ie, continuing care nursery), n = 3 (1.5%); Level 3 (ie, intermediate care nursery), n = 62 (30.5%); and Level 4 (ie, intensive care nursery), n = 137 (67.5%).

b Based on American Medical Association classification: Northeast: NJ, NY, PA, CT, ME, MA, NH, RI, VT; North Central: IL, IN, MI, OH, WI, IA, KS, MN, MO NE, ND, SD; South: AL, KY, MS, TN, DE, DC, FL, GA, MD, NC, SC, VA, WV, AR, LA, OK, TX; West: AZ, CO, ID, MT, NV, NM, UT, WY, AK, CA, HI, OR, WA.

c Responses may sum to >100%.

d Responses only available from the 107 neonatologists who completed the follow-up survey.

e CLDP for pediatric pulmonologists and HS-CHD for pediatric cardiologists.

### Administration of RSV Immunoprophylaxis

Nearly all physicians in these specialties (93.5%–98.7%) routinely recommended RSV immunoprophylaxis, even when poor parental compliance was suspected (Table 2). The most frequently provided reasons for not recommending RSV immunoprophylaxis varied slightly across specialties; overall, parental refusal and lack of or insufficient insurance overall were the reasons most commonly cited (Table 2).

**Table 2.** Reasons for Lack of RSV Immunoprophylaxis Recommendation.

	Neonatologists (n = 203)	Pediatricians (n = 138)	Pediatric Pulmonologists (n = 58)	Pediatric Cardiologists (n = 156)
Physicians recommending RSV immunoprophylaxis, even when poor parental compliance is suspected, n (%)	193 (95.1)	129 (93.5)	56 (96.6)	154 (98.7)
Most frequently selected reasons for lack of RSV immunoprophylaxis recommendation for high-risk children, n (%)^a^				
Parental refusal	81 (39.9)	77 (55.8)	41 (70.7)	85 (54.5)
Lack of/insufficient insurance	73 (36.0)	83 (60.1)	34 (58.6)	57 (36.5)
Perceived financial burden to the family	55 (27.1)	0	24 (41.4)	31 (19.9)
Inability of practice to manage prior authorization by the insurance company	52 (25.6)	29 (21.0)	—	—
Lack of demonstrated cost-effectiveness of RSV immunoprophylaxis	51 (25.1)	13 (9.4)	5 (8.6)	5 (3.2)
Contraindication/allergy to RSV immunoprophylaxis	36 (17.7)	45 (32.6)	26 (44.8)	74 (47.4)
Concerns regarding efficacy of RSV immunoprophylaxis	19 (9.4)	7 (5.1)	4 (6.9)	10 (6.4)
Burden of monthly injections	17 (8.4)	9 (6.5)	8 (13.8)	23 (14.7)
Perceived parental noncompliance	14 (6.9)	12 (8.7)	4 (6.9)	7 (4.5)
Concerns regarding safety of RSV immunoprophylaxis	4 (2.0)	2 (1.4)	1 (1.7)	3 (1.9)
Low platelet count	3 (1.5)	8 (5.8)	3 (5.2)	12 (7.7)
Other	39 (19.2)^b^	14 (10.1)^c^	5 (8.6)^d^	27 (17.3)^e^

Abbreviation: RSV, respiratory syncytial virus.

a Each respondent provided 3 response options.

b Other reasons include the following: all eligible children receive immunoprophylaxis (n = 35); continued return to location of sick contacts for immunization (n = 1); if insurance will cover cost (n = 2); and reluctance of managed care to approve for this age group in the presence of low risk (n = 1).

c Other reasons include the following: healthy infant clinically (n = 1); always recommend if necessary (n = 7); insurance denials (n = 2); and not applicable (n = 4).

d Other reasons include the following: child does not meet guidelines (n = 1); high risk is a variable term (n = 1); respondent does recommend RSV prophylaxis (n = 1); respondent noted that only Amish have refused prophylaxis; never had an allergy to any of the others (n = 1); and in the case where child was able to come off supportive care before 6 months in front of RSV season and <2 years old (n = 1).

e Other reasons include the following: respondent always recommends prophylaxis (n = 22); access to provider who administers RSV (n = 1); defer timing to primary care provider (n = 1); when child does not fit criteria (n = 1); family concerns regarding safety (n = 1); and insurance refusal to pay (n = 1).

The majority of respondents (87.2% of neonatologists, 83.3% of pediatricians, 91.4% of pediatric pulmonologists, and 71.8% of pediatric cardiologists) reported that >50.0% of eligible infants and young children received each recommended monthly dose of RSV immunoprophylaxis throughout the RSV season. In addition, most respondents (78.3% of neonatologists, 92.0% of pediatricians, 98.3% of pediatric pulmonologists, and 100% of pediatric cardiologists) would start monthly dosing for those who presented after the RSV season had begun (Table 3). Between 18.2% and 32.8% of all physicians surveyed had recommended more than 5 doses during the RSV season; this was most frequently attributed to a prolonged RSV season or ongoing prevalence of RSV in the community (Table 3). The proportion of respondents recommending RSV immunoprophylaxis for eligible young children in their second RSV season varied considerably by specialty, with a higher proportion of pediatric pulmonologists (94.8%) and pediatric cardiologists (95.5%) making such recommendations compared with a lower proportion of neonatologists (57.6%) and pediatricians (51.4%) (Table 3). However, among the pediatric pulmonologists and pediatric cardiologists who reported recommending RSV immunoprophylaxis in the second season, 65.5% and 67.8%, respectively, indicated that ≤30.0% of children who received it in their first season also received it in their second season.

**Table 3.** RSV Immunoprophylaxis Dosing Recommendations.

	Neonatologists (n = 203)	Pediatricians (n = 138)	Pediatric Pulmonologists (n = 58)	Pediatric Cardiologists (n = 156)
Recommendations for patients who present after the RSV season has begun, n (%)	n = 92^a^			
Starting dose followed by once a month until the end of season	72 (78.3)	127 (92.0)	57 (98.3)	156 (100)
No doses	5 (5.4)	2 (1.4)	0	0
Other	15 (16.3)^b^	9 (6.5)^c^	1 (1.7)^d^	0
Respondents ever recommending >5 doses of RSV immunoprophylaxis during a single RSV season, n (%)^c^	37 (18.2)	28 (20.3)	19 (32.8)	40 (25.6)
Respondents who recommend RSV immunoprophylaxis in the second RSV season to patients who received RSV immunoprophylaxis during the first season, n (%)	53 (57.6)^a^	71 (51.4)	55 (94.8)	149 (95.5)

Abbreviations: AAP, American Academy of Pediatrics; NICU, neonatal intensive care unit; RSV, respiratory syncytial virus; wGA, weeks’ gestational age.

a Options valid for neonatologists who manage preterm infants in a NICU follow-up clinic after discharge (n = 92).

b Other recommendations include the following: <32 wGA, 5 doses, 32 to 34 wGA, 3 doses (n = 1); 3 doses (n = 3); AAP criteria (n = 4); depends on current age (n = 2); depends on gestational age (n = 4); depends on risk factors (n = 2); depends on clinical factors (n = 1); and up to 5 doses (n = 1).

c Other recommendations include the following: as per guidelines (n = 1), 3 to 5 doses, depending on age (n = 5); depends on risk factors (n = 1); up to 3 months of age (n = 1); and until end of season (n = 1).

d Response includes the following: this is dictated by the insurance (n = 1).

Most neonatologists (98.0%), pediatricians (73.2%), pediatric pulmonologists (89.7%), and pediatric cardiologists (80.8%) reported that their primary hospital provides the first dose of RSV immunoprophylaxis before discharge from the birth hospitalization during the RSV season. However, for eligible infants who remain hospitalized for an extended period of time in the neonatal intensive care unit before the birth discharge, fewer than half of these neonatologists (34.7%) and pediatricians (43.6%) responded that their primary hospital provides additional monthly doses of RSV immunoprophylaxis throughout the RSV season (Table 4). All specialists reported that subsequent doses of RSV immunoprophylaxis administered in the outpatient setting were provided at a variety of locations, including the respondent’s office or clinic, through primary care providers or pediatricians, and at the patient’s home via a home health agency (Table 4).

**Table 4.** Administration of RSV Immunoprophylaxis: Location Where Doses Are Provided.

	Neonatologists (n = 203)	Pediatricians (n = 138)	Pediatric Pulmonologists (n = 58)	Pediatric Cardiologists (n = 156)
Respondents reporting that the primary hospital provides the first dose of RSV immunoprophylaxis before the birth discharge, n (%)	199 (98.0)	101 (73.2)	52 (89.7)	126 (80.8)
Primary hospital provides additional monthly doses of RSV immunoprophylaxis to eligible infants in the NICU throughout the RSV season before the birth discharge	69 (34.7)	44 (43.6)	—	—
Subsequent doses administered in each setting, when the primary hospital provides the first dose,^a^ mean percentage (SD)				
All subsequent doses administered at respondent’s office/clinic	25.3 (38.9)	73.0 (39.9)	48.3 (42.3)	28.3 (40.3)
Next dose administered at respondent’s office/clinic; remainder administered through PCP/pediatrician	19.4 (33.1)	6.4 (20.7)	14.0 (25.6)	16.1 (27.2)
Next dose administered at outpatient facility (either primary care or specialty care); remainder administered at home through a home health agency	25.4 (35.3)	30.8 (39.6)	16.9 (27.4)	22.8 (31.9)
All subsequent doses administered through PCP/pediatrician	69.0 (37.8)	36.3 (46.4)	60.2 (40.2)	76.2 (33.3)
All subsequent doses administered at home through a home health agency	26.3 (33.8)	22.1 (30.3)	26.0 (31.0)	22.5 (31.4)
Other	29.4 (47.0)^b^	43.1 (47.4)^c^	34.0 (47.7)^d^	75.0 (50.0)^e^

Abbreviations: NICU, neonatal intensive care unit; PCP, primary care provider; RSV, respiratory syncytial virus.

a Respondents could potentially select more than 1 response option, and rank in order of frequency.

b Other responses include the following: do not follow up with outpatients (n = 2); only work in NICU (n = 3); next and all subsequent doses administered through our academic pediatric department’s subspecialty care clinic, located in the same hospital (n = 1); and no other (n = 7).

c Other responses include the following: none (n = 7); based on insurance (n = 1); chronic care facility (n = 1); pediatric pulmonologist’s office (n = 2); pulmonary clinic (n = 2); special palivizumab clinic (n = 1); specialty clinic in other pediatric hospital (n = 1); and through vaccine coordinator (n = 1).

d Other responses include the following: not applicable or none (n = 3); neonatology high-risk outpatient clinic (n = 1); and hospital (n = 1).

e Other responses include the following: home nursing (n = 1) and pulmonology clinic (n = 3).

Most neonatologists (98.0%) identified themselves as the specialist primarily responsible for prescribing hospital-administered doses of RSV immunoprophylaxis. This was affirmed by most pediatricians (85.1%), who also reported that neonatologists were the physician type primarily responsible for prescribing hospital-administered doses of RSV immunoprophylaxis, followed by themselves (8.9%), pediatric pulmonologists (1.9%), and infectious disease specialists (1.9%). Pediatric pulmonologists also most frequently identified neonatologists as being primarily responsible for prescribing hospital-administered doses of RSV immunoprophylaxis (65.4%), followed by themselves (19.2%), and pediatricians (13.5%). In contrast, most pediatric cardiologists (59.5%) identified themselves as the specialist primarily responsible for prescribing hospital-administered doses of RSV immunoprophylaxis, followed by neonatologists (21.4%), and cardiac intensivists (8.7%).

### Communication Patterns Regarding RSV Immunoprophylaxis Administration

When RSV immunoprophylaxis is administered in the respondent’s clinic or office, most neonatologists, pediatricians, pediatric pulmonologists, and pediatric cardiologists (67.5% to 100%) reported scheduling a follow-up visit at the end of the current appointment to ensure receipt of subsequent doses (Table 5). Most (≥85.0%) respondents of all specialty types reported that they notified parents of missed appointments and that these reminders occurred most frequently by telephone. Moreover, even for doses they did not administer themselves, most neonatologists, pediatricians, and pediatric cardiologists reported that they notified parents of a missed follow-up visit, also primarily by telephone. Most pediatric pulmonologists (73.1%) and pediatric cardiologists (75.0%) reported using a facsimile or letter to communicate with primary care providers or pediatricians.

**Table 5.** Communication Patterns Regarding RSV Immunoprophylaxis Administration.

	Neonatologists	Pediatricians	Pediatric Pulmonologists	Pediatric Cardiologists
**For doses administered in the respondents’ clinic/office^a^**	n = 40	n = 98	n = 21	n = 30
Method ensuring return for follow-up visits to receive injection,^b^ n (%)				
Schedule the next injection at the end of the appointment for the current injection	27 (67.5)	89 (90.8)	21 (100)	24 (80.0)
Electronic reminders for parents (eg, email, text message)	11 (27.5)	32 (32.6)	6 (28.6)	12 (40.0)
Written reminders for parents by mail	19 (47.5)	22 (22.4)	4 (19.0)	12 (40.0)
Reminder for respondents (ie, tracking sheet)	7 (17.5)	35 (35.7)	5 (23.8)	7 (23.3)
Electronic health record pop-out	10 (25.0)	24 (24.5)	2 (9.5)	6 (20.0)
Other	6 (15.0)^c^	14 (14.3)^d^	5 (23.8)^e^	3 (10.0)^f^
Notification to parent of missed appointments				
No	6 (15.0)	1 (1.0)	1 (4.8)	2 (6.7)
Yes	34 (85.0)	97 (99.0)	20 (95.2)	28 (93.3)
By telephone^b^	29 (85.3)	95 (97.9)	19 (95.0)	26 (92.8)
By postcard/letter^b^	14 (41.2)	29 (29.9)	3 (15.0)	12 (42.9)
Electronic reminder (eg, email, text message)^b^	4 (11.8)	12 (12.4)	3 (15.0)	2 (7.1)
Other	1 (2.9)^g^	1 (1.0)^h^	0	0
Additional notification to PCP/pediatrician of missed appointments (among those who notified parent)	n = 34	n = 97	n = 20	n = 28
No	2 (5.9)	20 (20.4)	6 (30.0)	12 (42.9)
Yes	32 (94.1)	77 (69.6)	14 (70.0)	16 (57.1)
**For doses administered by the PCP/pediatrician or through a home health agency**			n = 52	n = 140
Method of communication between respondent and PCP/pediatrician,^b^ n (%)				
Fax/letter to PCP/pediatrician	—	—	38 (73.1)	105 (75.0)
Telephone/email to PCP/pediatrician	—	—	20 (38.5)	54 (38.6)
Combination of the above	—	—	19 (36.5)	60 (42.9)
Unified electronic medical record	—	—	17 (32.7)	51 (36.4)
Prescription provided to parents	—	—	7 (13.5)	10 (7.1)
Other	—	—	1 (1.9)^i^	8 (5.7)^j^
**For doses not administered in the respondents’ clinic/office^k^**	n = 52	n = 40	n = 37	n = 125
Method ensuring return for follow-up visits to check on medical status,^b^ n (%)				
Schedule a follow-up visit at the end of the current appointment	29 (55.8)	27 (6.5)	—	—
Written reminders for parents by mail	17 (32.7)	9 (22.5)	—	—
Electronic reminders for parents (eg, email, text message)	5 (9.6)	10 (25.0)	—	—
Reminder for respondent (ie, tracking sheet)	4 (7.7)	6 (15.0)	—	—
Electronic health record pop-out	3 (5.8)	4 (10.0)	—	—
Other	14 (26.9)^l^	6 (15.0)^m^	—	—
Parental notification of missed regular follow-up visit				
No	7 (13.5)	3 (7.5)	9 (24.3)	12 (9.6)
Yes	45 (86.5)	37 (92.5)	28 (75.7)	113 (90.4)
By telephone^b^	42 (93.3)	34 (91.9)	21 (75.0)	95 (84.1)
By postcard/letter^b^	22 (48.9)	18 (48.6)	12 (42.9)	69 (61.1)
By electronic reminder (eg, email, text message)^b^	2 (4.4)	9 (24.3)	5 (17.9)	14 (12.4)
Other	3 (6.7)^n^	1 (2.7)^o^	0	5 (4.4)^p^

Abbreviations: NICU, neonatal intensive care unit; PCP, primary care provider; RSV, respiratory syncytial virus.

a Reflects only respondents who administer doses at their office/clinic.

b Respondents could select multiple response options.

c Other responses include the following: no outpatient clinic (n = 1); does not provide immunization at office (n = 1); not applicable (n = 2); and phone call (n = 2).

d Other responses include the following: phone call (n = 12); contact patient/call or send a letter (n = 1); and homecare agency notifies (n = 1).

e Other response includes the following: phone call (n = 5).

f Other responses include the following: phone call (n = 2) and homecare agency (n = 1).

g Other method includes the following: does not provide immunization at office (n = 1).

h Other method includes the following: certified mail (n = 1).

i Other response includes the following: do not know (n = 1).

j Other responses include the following: communication with parents (n = 3); discharge summary (n = 1); not at respondent’s facility (n = 1); pharmacy communicates with pediatrician’s office (n = 2); and homecare agency (n = 1).

k Reflects only respondents who do not administer doses at their office/clinic.

l Other responses include the following: contact with PCP (n = 1); contact with pediatric subspecialty and family for next dose scheduled at current appointment (n = 1); respondent does not follow up (n = 1); follow-up with pediatrician (n = 1); respondent follows up at high-risk infant visits at 9 to 12 months of age, trusting pediatricians will ensure follow-up (n = 1); PCP’s or pediatrician’s responsibility (n = 5); NICU administration (n = 1); only ensure follow-up appointments (n = 1); registered nurse calls (n = 1); and visits in respondent’s high-risk follow-up clinic (n = 1).

m Other responses include the following: home health coordinates (n = 1); not applicable—inpatient only (n = 2); and phone call (n = 3).

n Other methods include the following: clinic coordinators send letter and phone (n = 1); developmental clinic only (n = 1); and telegram (n = 1).

o Other method includes the following: call center or registered nurse (n = 1).

p Other methods include the following: notify pediatrician (n = 2), email (n = 1); phone if chronic no show (n = 1); and involve social worker for chronic no shows (n = 1).

### Access to RSV Immunoprophylaxis: Obstacles and Methods for Facilitation

For all specialist groups surveyed, insurance denial was among the 3 most frequently cited obstacles to getting recommended RSV immunoprophylaxis doses to patients (Table 6). Limitation in commercial insurance coverage was also one of the 3 obstacles most frequently cited by neonatologists, pediatricians, and pediatric pulmonologists but not by pediatric cardiologists. Other frequently reported obstacles included unclear eligibility criteria for neonatologists, parental noncompliance and the perceived reliability/unreliability of the infant’s parents/caregivers for pediatricians, limitations in Medicaid coverage for pediatric pulmonologists, and parental noncompliance and communication gaps for pediatric cardiologists (Table 6).

**Table 6.** Access to RSV Immunoprophylaxis: Obstacles.

	Neonatologists (n = 203)	Pediatricians (n = 138)	Pediatric Pulmonologists (n = 58)	Pediatric Cardiologists (n = 156)
Biggest obstacles to getting RSV immunoprophylaxis to patients, n (%)^a^				
Insurance denials	73 (36.0)	80 (58.0)	30 (51.7)	54 (34.6)
Unclear eligibility criteria	26 (12.8)	4 (2.9)	2 (3.4)	15 (9.6)
Limitations to commercial insurance coverage	24 (11.8)	12 (8.7)	12 (20.7)	11 (7.1)
Limitations to Medicaid coverage	16 (7.9)	7 (5.1)	1 (1.7)	9 (5.8)
Noncompliance	14 (6.9)	14 (10.1)	5 (8.6)	24 (15.4)
Reliability of parents/caretakers	12 (5.9)	8 (5.8)	2 (3.4)	7 (4.5)
Communication gaps between hospital discharge team and prescriber	12 (5.9)	3 (2.2)	4 (6.9)	16 (10.3)
Communication gaps between PCP and specialist	7 (3.4)	1 (0.7)	1 (1.7)	5 (3.2)
Respondent forgets	4 (2.0)	5 (3.6)	1 (1.7)	9 (5.8)
Child’s family stability	4 (2.0)	3 (2.2)	0	4 (2.6)
Other	11 (5.4)^b^	0	0	2 (1.3)^c^
Three biggest obstacles to getting RSV immunoprophylaxis to patients, n (%)^d^				
Insurance denials	118 (58.1)	95 (68.8)	39 (67.2)	79 (50.6)
Limitations to commercial insurance coverage	87 (42.9)	65 (47.1)	29 (50.0)	55 (35.2)
Unclear eligibility criteria	73 (36.0)	37 (26.8)	20 (34.5)	38 (24.3)
Noncompliance	69 (34.0)	52 (37.7)	13 (22.4)	65 (41.7)
Reliability of parents/caretakers	61 (30.0)	49 (35.5)	18 (31.0)	50 (32.0)
Limitations to Medicaid coverage	59 (29.1)	49 (35.5)	21 (36.2)	35 (22.4)
Communication gaps between PCP and specialist	38 (18.7)	16 (11.6)	11 (19.0)	57 (36.5)
Child’s family stability	33 (16.3)	23 (16.7)	5 (8.6)	20 (12.8)
Communication gaps between hospital discharge team and prescriber	33 (16.2)	16 (11.6)	14 (24.1)	40 (25.6)
Respondent forgets	18 (8.9)	9 (6.5)	3 (5.1)	25 (16.0)
Other	19 (9.3)^b^	2 (1.4)^e^	1 (1.7)^f^	2 (1.3)^c^

Abbreviations: PCP, primary care provider; RSV, respiratory syncytial virus.

a This section tabulates the top-ranked obstacles.

b Other obstacles include the following: current guidelines (n = 2), all of our patients receive prior to discharge (n = 1), cost (n = 1), insurance approval (n = 1), medical issues (n = 1), no issues (n = 10), patient does not qualify (n = 1), refusal to vaccinate (n = 1), and surprise discharge before being able to give first dose (n = 1).

c Other obstacles include the following: family practice offices not providing dose (n = 1), high cost (n = 1), and parent’s misconception about safety of vaccination (n = 1).

d This section tabulates the top 3 ranked obstacles.

e Other obstacles include the following: physician apathy (n = 1) and verbose eligibility criteria (n = 1).

f Other obstacle includes the following: hospital administration (n = 1).

Respondents of all specialties thought that checklists, electronic reminders, and additional education and training were valuable tools to facilitate provision of RSV immunoprophylaxis. One of the most frequently cited methods across all respondent types was written checklists or “cheat sheets” reminding respondents of the eligibility criteria (neonatologists, 22.2%; pediatricians, 30.4%; pediatric pulmonologists, 17.2%; and pediatric cardiologists, 16.0%). Electronic health record pop-up reminders were cited by 23.2% of neonatologists, 24.1% of pediatric pulmonologists, and 35.3% of pediatric cardiologists. Additionally, more training or seminars specific to risk factors provided to neonatologists and primary care providers were cited by 16.7% of neonatologists and pediatricians and 21.8% of pediatric cardiologists as a method to facilitate provision of RSV immunoprophylaxis. Pediatricians (18.8%) and pediatric pulmonologists (27.6%) highlighted insurance letter templates as another valuable tool to help ensure that high-risk children receive RSV immunoprophylaxis.

## Discussion

To date, the available literature regarding the provision of RSV immunoprophylaxis in the clinical setting has been limited. This study identified the logistics of, and barriers to, RSV immunoprophylaxis administration and communication patterns surrounding its use among neonatologists, pediatricians, pediatric pulmonologists, and pediatric cardiologists. Recognition of the barriers to RSV immunoprophylaxis access and administration as well as identification of strategies to improve access and communication between physicians and patients’ parents are important steps to enhance adherence to RSV immunoprophylaxis, and ultimately improve RSV disease outcomes.

Previous studies have demonstrated the importance of RSV immunoprophylaxis in reducing the risk of hospitalizations caused by severe RSV disease among infants at high risk.^3,4^ However, each dose of RSV immunoprophylaxis only provides protection from severe RSV disease for approximately 1 month; thus, the first dose should be administered at the start of the RSV season and the remaining doses administered monthly throughout the RSV season.^9^ In the present study, a high proportion of all survey respondents reported that their primary hospital administers the first dose of immunoprophylaxis to infants before discharge from the birth hospitalization during the RSV season. This is beneficial because a delay in initiating immunoprophylaxis places susceptible infants at risk for developing severe RSV disease. In a study by Speer et al,^10^ among infants who received their initial dose of RSV immunoprophylaxis in the outpatient setting from 2000 to 2004, the mean time to receipt of the first dose was 26 days (range, 1 day to >4 months) after discharge. Administration of the first dose before hospital discharge emphasizes its importance to parents and provides an opportunity to discuss the necessity of monthly doses. Assistance with scheduling follow-up visits can also facilitate compliance and adherence with timely receipt of subsequent doses. A small proportion of respondents indicated that the first dose of immunoprophylaxis is not administered by their primary hospital. Some possibilities include a hospital policy against predischarge dosing or the lack of routine identification of infants eligible to receive RSV immunoprophylaxis.

In the present study, respondents reported that subsequent outpatient doses of RSV immunoprophylaxis are administered by a variety of health care providers, most commonly by primary care providers or pediatricians. However, a large proportion of young children with CLDP and HS-CHD receive their doses in the pediatric pulmonologists’ and pediatric cardiologists’ office or clinic. The varied providers and settings in which immunoprophylaxis is administered underscores the importance of ongoing collaborative efforts to coordinate patient care. Regular communication and partnership among specialists will help ensure that all eligible preterm infants and young children at high risk for severe RSV disease receive immunoprophylaxis throughout the entire RSV season without gaps in the monthly dosing schedule.

Parental understanding of the importance of immunoprophylaxis is essential to ensure that each recommended monthly dose is administered in a timely manner. It has been previously shown that the strongest association of compliance with the receipt of all doses is parents’ perception of the benefits of RSV immunoprophylaxis.^11^ Therefore, counseling parents on the purpose of RSV immunoprophylaxis, how it works, and the importance of adherence is also critical.^12,13^ Parents should be educated about the fact that RSV immunoprophylaxis provides passive immunization rather than eliciting an active immune response against RSV; therefore, immunoprophylaxis must be administered monthly throughout the RSV season for it to be most effective. Furthermore, providing re-education at each follow-up visit regarding the mechanism of protection and the importance of monthly doses to derive the full benefits of protection would be expected to bolster adherence to the dosing schedule and help prevent RSV-related hospitalizations. It is also important to address the fears and concerns of parents regarding possible side effects of immunoprophylaxis and to consider cultural backgrounds, religious beliefs, literacy, and language skills when counseling them.

An alternative setting in which to receive RSV immunoprophylaxis that has been shown to improve compliance is in the home, with the added benefit of convenience to the family. In a study of 1446 infants, 224 infants received the recommended RSV immunoprophylaxis at the pediatrician’s office, and 969 received RSV immunoprophylaxis at home by a nurse every month.^14^ More infants who received doses at home (98.0%) had subsequent doses given on schedule compared with those who were given doses in the pediatrician’s office (89.2%; *P* < 0.001).^14^ Furthermore, there was a significantly higher percentage of infants in the office setting group with an RSV-related hospitalization compared with those who received doses in the home setting (3.57% vs 0.93%; *P* < 0.001).^14^ Although findings from this study suggest better adherence with home RSV immunoprophylaxis injections and a lower rate of RSV-related hospitalization as a result, it is important to understand that home-based therapeutic plans may not be practical to implement everywhere and that the need for insurance approval may delay timely receipt of immunoprophylaxis.

In terms of access to RSV immunoprophylaxis, survey respondents of all specialties reported insurance denials as the primary obstacle in getting RSV immunoprophylaxis to high-risk children. Health insurance companies consider several factors when determining coverage for RSV immunoprophylaxis. Because RSV immunoprophylaxis is only indicated for certain at-risk infants and must be administered during a particular time of the year (ie, the RSV season), most insurance companies require prior authorization.^15^ It is important that pediatricians and other specialists are aware of, and educated about, the process of obtaining prior authorization and that this process begins promptly for at-risk children who are eligible and recommended for RSV immunoprophylaxis.

Health insurance companies can assist in improving compliance through the use of specialty pharmacies that provide access to RSV immunoprophylaxis.^15^ In addition to providing the medication, specialty pharmacists can also counsel parents and assist with scheduling follow-up appointments.^15^ Moreover, specialty pharmacies can collaborate with home care agencies to administer RSV immunoprophylaxis in the patient’s home, which may further improve adherence.^15^

A strength of this study is that respondents for the survey were selected from the entire pool of AMA-registered physicians. Their characteristics are representative of the overall target population.^16^ Additionally, all individuals in the AMA physician master file who self-identified as neonatologists, pediatric pulmonologists, or pediatric cardiologists as well as a randomly selected sample of pediatricians received the survey, maintaining generalizability and reducing the potential for selection bias. Response bias is an inherent limitation of survey studies and may have affected the findings from this study because the respondents of the survey may be more interested in RSV immunoprophylaxis than those who did not respond. It is possible that their responses may not be reflective of the general population of practicing physicians of these specialty types.

## Conclusions

The findings from this study suggest that the vast majority of surveyed US neonatologists, pediatricians, pediatric pulmonologists, and pediatric cardiologists routinely recommend RSV immunoprophylaxis for high-risk children and that eligible infants receive their first dose before being discharged from the birth hospitalization during the RSV season. Adherence with subsequent dosing may be improved by heightened collaboration between neonatologists, pediatricians, and subspecialists in the outpatient setting as part of a coordinated, multidisciplinary approach that focuses on education and follow-up strategies. In addition, addressing the practical issues of interpretation of eligibility criteria and insurance coverage are likely to facilitate the use of RSV immunoprophylaxis in high-risk patients.

## Author Contributions

All authors contributed to the design, analysis, and interpretation of the study, critically revised the manuscript, provided final approval, and agreed to be accountable for all aspects of the work ensuring integrity and accuracy. DMF, KKM, and VRK contributed to the conception of the study. IAS, SMS, KKM, and VRK also contributed to the acquisition of data and drafted the manuscript.

## Supplementary Material

Supplementary material
