# Supplementary material for: The Logistics and Coordination of Respiratory Syncytial Virus Immunoprophylaxis Use Among US Pediatric Specialists
Source: Clin Pediatr (Phila). 2016 Jan 8;55(13):1230–41. doi: 10.1177/0009922815621343 (PMC5119619; doi:10.1177/0009922815621343)

Appendix I

Screener and Survey for Pediatricians and Neonatologists

Screener

1. Please indicate:
    a. The geographic location of your practice *(Options: drop-down menu with state names)(proceed to next question)*
    b. Your sex (*Options: male; female) (proceed to next question)*
    c. Your specialty *(Options: Neonatology; Pediatrics; None of the above) (if "None of the above," screener ends; otherwise, proceed to next question)*
    d. Considering the sample of infants in your practice with characteristics that put them at high risk for severe respiratory syncytial virus (RSV) disease, do you recommend RSV immunoprophylaxis with Synagis® (palivizumab)? *(Options: yes; no) (if "no," proceed to question 1.d.i., after which the participant is not eligible to continue with survey; if "yes," proceed to question 1.d.ii., then proceed to survey)*
        i. Why? *(open ended)*
        ii. Please provide the approximate number of patients for whom you have recommended RSV immunoprophylaxis in the past 12 months? *(open ended)*

Section 1: Respondent/Practice Information

1. Please indicate your
    a. Year of graduation *(If "Neonatology" selected for question Screener 1.c., then proceed to 1.a.i.; if "Pediatrics" selected for question Screener 1.c., then proceed to 1.a.ii)*
        i. From fellowship *(open ended)*
        ii. From residency *(open ended)*
    b. Practice type (*Options: medical school–based teaching hospital; non–medical school teaching hospital; nonteaching community hospital; large group practice (≥5 pediatric specialists); small group practice (<5 pediatric specialists); or individual practice*
    c. Primary area of practice (*Options (select as many as apply): Neonatal Intensive Care Unit (NICU); Inpatient; Outpatient)*

2. *If the response to Screener 1.c. was "Pediatrics," proceed to question 2.a. then 3.; if the response to Screener 1.c. was "Neonatology," proceed to questions 2.b., 2.c., and 2.d. then 3.)*
    a. What type of community does your primary practice serve? *(Options [select as many options as relevant]: Inner city; Urban [non–inner city]); Suburban; Rural)*
    b. Do you practice in more than one NICU? *(Options: Yes; No)*

c. What is the highest level nursery you service? *(Options: Level I = normal care nursery; Level II = continuing care nursery; Level III = intermediate care nursery; Level IV = intensive care nursery)*
d. Do you manage preterm infants in a neonatal follow-up clinic after discharge from the birth hospitalization? *(Options: Yes; No)*

3. Considering all preterm infants (12 months or younger) within your practice who were born at ≤35 weeks' gestational age (GA) without chronic lung disease (CLD) or hemodynamically significant congenital heart disease (CHD), please indicate *(responses to 3.b., 3.c., and 3. d. should total 100%)*
   a. The number for whom you have cared in the past 12 months *(Open ended)*
   b. The proportion of those for whom you cared in the past 12 months who were born at <29 weeks' GA *(Open ended)*
   c. The proportion of those for whom you cared in the past 12 months who were born at 29 to 31 weeks' GA *(Open ended)*
   d. The proportion of those for whom you cared in the past 12 months who were born at 32 to 35 weeks' GA *(Open ended)*

Section 2: Current Practice for Preterm Infants Born at <32 Weeks' GA (at or Before 31 Weeks, 6 Days of Gestation)

4. The 2012 American Academy of Pediatrics (AAP) guidelines for RSV immunoprophylaxis state: "Infants in this category may benefit from RSV prophylaxis, even if they do not have CLD [chronic lung disease]. For these infants, major risk factors to consider include gestational age and chronologic age at the start of the RSV season. Infants born at or before 28 weeks, 6 days' gestation may benefit from prophylaxis during the RSV season, whenever that occurs during the first 12 months of life. Infants born at 29 weeks, 0 days through 31 weeks, 6 days of gestation may benefit most from prophylaxis up to 6 months of age. However, once an infant qualifies for initiation of prophylaxis at the start of the RSV season, administration should continue throughout the season and should not stop when the infant reaches either 6 months or 12 months of age. A maximum of 5 monthly doses is recommended for infants in this category."

   By gestational age considered below, among those eligible during the most recent RSV season, please provide the approximate percentage of preterm infants without CLD or CHD for whom you recommended RSV immunoprophylaxis and the percentage who received RSV immunoprophylaxis. Also, please rate the clinical need for RSV immunoprophylaxis in the specific population.

	**<u>Among those eligible during the RSV season,</u> proportion (%) for whom RSV**	**<u>Among those eligible during the RSV season,</u> proportion (%) that received at**	**Clinical need for RSV immunoprophy-laxis (1–5 scale, 1= no need, 5=**

	**immunoprophy-laxis was recommended**	**least one dose of RSV immunoprophy-laxis**	**very high need)**
If infant was born:			
At or before 28 weeks, 6 days' GA and was <6 months of age at the start of the RSV season			
At or before 28 weeks, 6 days' GA and was 6 to <12 months of age at the start of the RSV season			
At 29 weeks, 0 days through 31 weeks, 6 days GA and was <6 months of age at the start of the RSV season			
At 29 weeks, 0 days through 31 weeks, 6 days GA and was 6 to <12 months of age at the start of the RSV season			

Section 3: Current Practice for Preterm Infants Born at 32 to Less Than 35 Weeks' Gestation (Defined as 32 Weeks, 0 Days Through 34 weeks, 6 Days of Gestation)

5. The 2012 AAP guidelines for RSV immunoprophylaxis state: "Numerous factors have been proposed as increasing the risk of acquiring RSV infection among infants in this gestational age group. Other factors have been associated with an increased risk of severe disease and hospitalization. Certain factors (CHD [congenital heart disease], prematurity, CLD) are well-established risk factors for hospitalization, because they consistently are present across numerous studies. In contrast, other reported risk factors either are found inconsistently, even in studies by the same authors, or increase the risk of hospitalization by a relatively small factor (less than twofold to threefold). A risk-scoring tool developed from a Canadian prospective study of infants born at 33 through 35 weeks' gestation revealed that multiple risk factors needed to be present before a significant increase in hospitalization risk was seen. Available data do not allow for definition of a subgroup of infants who are at risk of prolonged hospitalization and admission to the intensive care unit. Therefore, although current recommendations were designed to be consistent with the US Food and Drug Administration's approval for marketing of palivizumab for the prevention of serious RSV lower respiratory tract disease, they specifically target infants in this group with consistently identified risk factors for RSV hospitalization during the period of greatest risk, which is the first 3 months of life. Palivizumab prophylaxis should be limited to infants in this group at greatest risk of hospitalization attributable to RSV infection, namely infants younger than 3 months of age at the start of the RSV season or

who are born during the RSV season and who are likely to have an increased risk of exposure to RSV. Epidemiologic data suggest that RSV infection is more likely to occur and more likely to lead to hospitalization for infants in this gestational age group when at least 1 of the following 2 risk factors is present:

- The infant attends child care, defined as a home or facility where care is provided for any number of infants or young toddlers; or
- One or more older siblings younger than 5 years of age or other children younger than 5 years of age lives permanently in the same household. Multiple births younger than 1 year of age do not qualify as fulfilling this risk factor.

Prophylaxis may be considered for infants born at 32 through less than 35 weeks' gestation (defined as 32 weeks, 0 days through 34 weeks, 6 days of gestation) who are born less than 3 months before the onset of or during the RSV season and for whom at least 1 of the 2 risk factors is present. Infants in this gestational age category should receive prophylaxis only until they reach 3 months of age and should receive a maximum of 3 monthly doses; many will receive only 1 or 2 doses before they reach 3 months of age. Once an infant has passed 90 days of age, the risk of hospitalization attributable to RSV lower respiratory tract disease is reduced. Administration of palivizumab is not recommended after 3 months of age for patients in this category."

By gestational age considered below, please provide the approximate percentage of preterm infants without CLD or CHD for whom you recommended RSV immunoprophylaxis and the percentage who received RSV immunoprophylaxis, during the most recent RSV season. Also, please rate the clinical need for RSV immunoprophylaxis in the specific population.

	**During the RSV season, proportion (%) for whom RSV immunopro-phylaxis was recommended**	**During the RSV season, proportion (%) that received at least one dose of RSV immunopro-phylaxis**	**Clinical need for RSV immunopro-phylaxis (1–5 scale, 1= no need, 5= very high need)**
If infant was born at:			
32 weeks, 0 days through 34 weeks, 6 days GA and was <3 months of age at the start of the RSV season and has			
Neither of the 2012 AAP risk			

factors			
At least one of the 2012 AAP risk factors			
32 weeks, 0 days through 34 weeks, 6 days GA and was 3 to <6 months of age <u>at the start of the RSV</u> season and has			
Neither of the 2012 AAP risk factors			
At least one of the 2012 AAP risk factors			
35 weeks, 0 days through 35 weeks, 6 days GA and was <3 months of age <u>at the start of the RSV season</u> and has			
Neither of the 2012 AAP risk factors			
At least one of the 2012 AAP risk factors			
35 weeks, 0 days through 35 weeks, 6 days GA and was 3 to <6 months of age <u>at the start of the RSV season</u> and has			
Neither of the 2012 AAP risk factors			
At least one of the 2012 AAP risk factors			

6. Would you <u>recommend</u> RSV immunoprophylaxis for preterm infants born at ≤35 weeks' GA without CLD or CHD, whom you identify as being at high risk for severe RSV disease <u>even if you suspect there would be poor parental compliance with the recommendation</u>? (*Options: Yes, I would recommend it despite my suspicion of poor compliance; No, I would not recommend it because of anticipated poor compliance).*

7. If you do not recommend RSV immunoprophylaxis for all eligible high-risk preterm infants born at ≤35 weeks' GA without CLD or CHD, what are the top <u>three most common reasons</u> for a lack of recommendation? *(Options include the following: allow for selection of up to three answers)*
    a. *Contraindication or allergy to RSV immunoprophylaxis*
    b. *Low platelet count*
    c. *Burden of monthly injections*
    d. *Perceived parental noncompliance*
    e. *Parental refusal*
    f. *Lack of or insufficient insurance*
    g. *Perceived financial burden to the family*
    h. *Lack of demonstrated cost-effectiveness of RSV immunoprophylaxis*
    i. *Concerns regarding efficacy of RSV immunoprophylaxis*

j. *Concerns regarding safety of RSV immunoprophylaxis*
k. *Inability of the practice to manage prior authorization by the insurance company*
l. *Other (please specify)*

8. Of all preterm infants born at ≤35 weeks' GA without CLD or CHD to whom you recommend RSV immunoprophylaxis and who receive at least one dose, approximately what percentage of infants in your practice receive each recommended monthly dose throughout the RSV season? *(Open-ended)*

9. (*If the response to Screener 1c was "Pediatrics," OR if the response to Screener 1c was "Neonatologist" and response to 2d was "yes," then proceed to question 9, else go to question 10)* A preterm infant ≤35 weeks' GA without CLD or CHD who is eligible for RSV immunoprophylaxis arrives at your office or clinic and the RSV season has already begun. How many doses would you recommend that this infant receive? (*Options: starting dose followed by once a month until end of season; no doses; other (please specify)*

10. Do you ever recommend or prescribe more than 5 doses of RSV immunoprophylaxis during a single RSV season to a preterm infant ≤35 weeks' GA without CLD or CHD? *(Options: yes; no) (If 'yes', proceed to question 10a, otherwise, proceed to question 11)*
    a. Under what circumstances? *(open-ended)*

(*If the response to Screener 1c was "Pediatrics," OR if the response to Screener 1c was "Neonatologist" and response to 2d was "yes," then proceed to question 11, else go to question 12)*

11. Of the preterm infants ≤35 weeks' GA without CLD or CHD for whom you recommend RSV immunoprophylaxis in their first RSV season, do you recommend that they receive it in their second RSV season if they are still eligible based on their age at the start of the second RSV season? *(Options: yes, no) (If 'no,' proceed to question 12, if 'yes,' proceed to question 11a, then 11b, and then 12)*
    a. Under what circumstances? *(open-ended)*
    b. Approximately what percentage of those who receive RSV immunoprophylaxis in their first RSV season continue to receive it in their second RSV season? *(open-ended)*

Section 4: Administration of RSV Immunoprophylaxis

12. Does the primary hospital with which you're affiliated provide a dose of RSV immunoprophylaxis to eligible infants prior to the birth discharge?
    a. Yes, the primary hospital with which I am affiliated provides a dose of RSV immunoprophylaxis prior to discharge. (*go to question 13, then 14, then 15)*

b. No, the primary hospital with which I am affiliated does not provide a dose of RSV immunoprophylaxis prior to discharge. (*go to question 16)*
c. I am not affiliated with any specific hospital (*go to question 16)*

13. In addition to any doses provided immediately prior to discharge, does the primary hospital with which you're affiliated provide additional monthly doses of RSV immunoprophylaxis to eligible infants in the NICU throughout the RSV season (ie, weeks/months prior to discharge)?
    a. Yes, the primary hospital with which I am affiliated provides additional monthly doses of RSV immunoprophylaxis during the RSV season.
    b. No, the primary hospital with which I am affiliated does not provide additional monthly doses of RSV immunoprophylaxis during the RSV season.
    c. I am not affiliated with any specific hospital

14. If the primary hospital with which you are affiliated provides a dose of RSV immunoprophylaxis prior to discharge, how are the remaining doses administered? *Responses should add up to 100%.*
    a. All subsequent doses administered at your office or clinic *(Numeric)*
    b. The next dose administered at your office or clinic and the remainder through the primary care provider/pediatrician's office *(Numeric)*
    c. The next dose administered at an outpatient facility (either primary care or neonatology); with the outpatient facility making arrangements for subsequent doses to be administered by a home health agency *(Numeric)*
    d. All subsequent doses administered through the primary care provider/pediatrician. *(Numeric)*
    e. All subsequent doses administered at home by home health agency *(Numeric)*
    f. Other *(please specify)*

15. For hospital-administered doses, which specialist is typically responsible for prescribing RSV immunoprophylaxis to your preterm patients born at ≤35 weeks' GA without CLD or CHD? *(Options [rank the responses in order of frequency if more than one response option applies]: Neonatologist; Pediatric Pulmonologist; Pulmonology intensivist; Infectious disease specialist; Pediatrician; don't know)*

16. If the primary hospital with which you are affiliated does not provide a dose of RSV immunoprophylaxis to your preterm patients born at ≤35 weeks' GA without CLD or CHD prior to discharge, or if you are not affiliated with a hospital, how do infants for whom you recommend RSV immunoprophylaxis receive it? *Responses should add up to 100%.*
    a. All doses administered at your office or clinic *(Numeric)*
    b. First dose administered at your office or clinic; subsequent doses administered through the primary care provider/pediatrician *(Numeric)*

c. First dose administered at an outpatient facility (either primary care or neonatology); with the outpatient facility making arrangements for subsequent doses to be administered by a home health agency *(Numeric)*
d. All doses administered through the primary care provider/pediatrician *(Numeric)*
e. All doses administered at home by home health agency *(Numeric)*

(If the response to Screener 1c was "Pediatrics", OR if the response to Screener 1c was "Neonatologist" and response to 2d was "yes", then question 17, 18, 19 and 20 may apply; else, proceed to question 21)

17. *(This question will only appear if 14a, 14 b, 16a and/or 16b are selected)* For doses that are administered at your office or clinic, how do you assure that the infant comes back for follow-up visits to receive their RSV immunoprophylaxis injection? *(Options [select as many as apply]):*
    a. Electronic reminders for parents (eg, email, text message)
    b. Written reminders for parents by mail
    c. Reminder for yourself (eg, tracking sheet)
    d. Electronic health record pop-out
    e. Schedule the next RSV immunoprophylaxis injection at the end of the appointment for the current injection
    f. Other *(please specify)*

18. *(This question will only appear if 14a, 14b, 16a and/or 16b are selected)* For doses that are administered at your office or clinic, if an infant misses an appointment to receive a RSV immunoprophylaxis injection, do you notify their parent(s) of their missed appointment? *(Options: yes; no) (If "yes," then proceed to question 18a and then 18b, else go to question 19)*
    a. How do you notify them? *(select as many as apply)*
        i. *Telephone*
        ii. *Postcard/letter*
        iii. *Electronic reminder (eg, email, text message)*
        iv. *Other (please specify)*
    a. Would you also inform the infant's primary care provider of the missed dose? *(Options: yes; no)*

19. *(This question will only appear if 14a,14b,16a, and/or 16b NOT are selected)* If the RSV immunoprophylaxis doses are not administered at your office or clinic, how do you assure that the infant comes back for follow-up visits with you to check on their medical status? *(Options include the following: select as many as apply)*
    a. Electronic reminders for parents (eg, email, text message)
    b. Written reminders for parents, by mail
    c. Reminder for yourself (eg, tracking sheet)
    d. Electronic health record pop-up
    e. Schedule a follow-up visit at the end of the current visit

f. Other *(please specify)*

20. *(This question will only appear if 14a,14b,16a, and/or 16b NOT are selected)* If an infant misses a regular follow-up visit with you regarding their medical status, do you notify their parent(s) of their missed appointment? *(Options: yes; no) (If 'yes,' then proceed to question 20a, otherwise, proceed to question 21)*
    a. How do you notify them? *(select as many as apply)*
        i. *Telephone*
        ii. *Postcard/letter*
        iii. *Electronic reminder (eg, email, text message)*
        iv. *Other (please specify)*

Section 5: Access to RSV Immunoprophylaxis

21. Are there any patient groups for whom you think RSV immunoprophylaxis should be provided but is not currently recommended by the 2012 AAP guidelines? *(Options: yes; no) (If "yes," then proceed to question 21a, otherwise proceed to question 22)*
    a. Which groups? *(Options [select as many as apply]: those with early congestive heart failure; those expected to have surgery during the RSV season; those anticipated to develop CHD; those who have Down syndrome without congenital heart disease; those who have Down syndrome with congenital heart disease; those with cystic fibrosis; those with congenital anomalies of the respiratory system; those with congenital diaphragmatic hernia; those with history of mechanical ventilation; those with history of meconium aspiration; other [please specify])*

22. What is the biggest obstacle in getting RSV immunoprophylaxis to your preterm patients born at ≤35 weeks' GA without CLD or CHD? *(Options (rank top three): you forget; insurance denials; limitations to commercial insurance coverage; limitations to Medicaid coverage; unclear eligibility criteria; nonadherence; communication gaps between hospital discharge team and prescriber; communication gaps between primary care provider and neonatologist; infant's family stability; reliability of infant's parents/caretakers; other [please specify])*

23. What would be the biggest help to you to facilitate provision of RSV immunoprophylaxis to your preterm patients born at ≤35 weeks' GA without CLD or CHD? *(Options [rank top 3]: more training or seminars provided to neonatologists and primary care providers specific to risk factors; written checklists reminding you of the eligibility criteria; electronic health record pop-up reminders; detail sales representatives; quality improvement modules specific for RSV immunoprophylaxis; insurance letter templates; cost-effectiveness data; other [please specify])*

24. In your opinion, what is the best tool you could have to ensure that eligible infants get RSV immunoprophylaxis? *(Options [rank top three]: Electronic health record pop-up reminders; neonatologist and primary care provider involvement in the discharge planning process; practice-based reminder schedule on paper or computer; reminder at each infant encounter by sticker or electronic; your own memory; parental education prior to discharge; other [please specify])*

25. Would you like to receive an honorarium for completing the survey? *(Options: yes, no) (If "no," proceed to question a, if "yes,"' proceed to question b)*
    - *a.* Thank you very much for participating in the survey.
    - *b.* Which of the following do you prefer? *(only allow participant to select one of the following)*
    - i. A $75 Amazon voucher *(Options: yes, no) (If "yes," ask for the following)*

      Your name *(open-ended)*

      Your work address *(open-ended)*

      Your email address *(open-ended)*
    - ii. Donation of $75 to UNESCO *(Options: yes, no) (If "yes," guide them for donation)*

Appendix II

Screener and Survey for Pediatric Pulmonologists

Screening

1. Please indicate:
   a. The geographic location of your practice *(Options: drop-down menu with state names)(proceed to next question)*
   b. Your sex (*Options: male; female) (proceed to next question)*
   c. Your specialty *(Options: Pediatric Pulmonology; Adult Pulmonology; None of the above) (if "Adult Pulmonology" or "None of the above," screener ends, else proceed to next question)*
   d. Considering the sample of children in your practice with characteristics that put them at high risk for severe respiratory syncytial virus (RSV) disease, do you recommend RSV immunoprophylaxis? *(Options: yes; no) (if "no," proceed to question 1di, after which the participant is not eligible to continue with survey, if '"yes," proceed to question 1dii then proceed to survey)*
      i. why? *(open-ended)*
      ii. please provide the approximate number of patients for whom you have recommended RSV immunoprophylaxis in the past 12 months? *(open-ended)*

Section 1: Respondent/Practice Information

1. Please indicate your:
   a. Year of graduation from fellowship *(open-ended)*
   b. Primary practice type (*Options: medical school-based teaching hospital; non–medical school teaching hospital; non–teaching community hospital; large group practice (≥5 pediatric specialists); small group (<5 pediatric specialists); or individual practice)*
   c. Primary area of pediatric pulmonology (*Options (select as many as apply): Outpatient; Intensive care unit; Inpatient ward; Research)*

2. What type of community does your primary practice serve? *(Options [select as many options as relevant]: Inner city; Urban [non–inner city]); Suburban; Rural)*

3. Please indicate the approximate number of children (24 months of age or younger) within your practice with chronic lung disease of prematurity (CLDP):
   a. Whom you cared for in the past 12 months *(Open-ended)*

b. The proportion of those whom you cared for in the past 12 months, who received RSV immunoprophylaxis in the past 12 months *(Options: <20%; 20%–39%; 40%–59%; 60%–79%; ≥80%)*

Section 2: Current Practice

4. The 2012 American Academy of Pediatrics (AAP) guidelines for RSV immunoprophylaxis state: "Palivizumab prophylaxis may be considered for infants and children younger than 24 months of age who receive medical therapy (supplemental oxygen, bronchodilator, diuretic or chronic corticosteroid therapy) for CLD within 6 months before the start of the RSV season. These infants and young children should receive a maximum of 5 doses. Patients with the most severe CLD who continue to require medical therapy may benefit from prophylaxis during a second RSV season."

   For each factor considered below, please provide the approximate percentage of children with CLDP (24 months of age or younger at start of RSV season) that you would expect to have the specified condition, in your practice. Please also identify if the following factors are indicative of: a) CLDP; b) high risk for severe RSV disease, and/or c) eligibility for RSV immunoprophylaxis?

	**Expected prevalence (%) among children with CLDP in your practice** ***(open-ended)***	**Indicative, in whole or in part, of CLDP (Options Y/N)**	**At high risk for severe RSV disease** ***(Options: Y/N)***	**Eligible for RSV immuno-prophylaxis** ***(Options: Y/N)***
Considering a child, at the start of the RSV season:				
Is receiving medical therapy (ie, supplemental oxygen, bronchodilator, diuretic, and/or corticosteroid)				
Currently has resting tachypnea or respiratory distress				
Currently has hypoxemia				
Currently has feeding difficulties due to breathing problems				
Currently has poor growth or				

poor weight gain due to breathing problems				
Currently has gastroesophageal reflux and breathing difficulties				
With prior severe respiratory illness (requiring intensive care)				
With prior severe respiratory illness (requiring inpatient, non–intensive care)				
With prior mild-moderate respiratory illness (requiring outpatient provider visit)				
With other comorbid cardiac conditions				

	**Expected prevalence (%) <u>among children with CLDP in your practice</u>** ***(open-ended)***	**Indicative, in whole or in part, of CLDP (Options Y/N)**	**At high risk for severe RSV disease** ***(Options: Y/N)***	**Eligible for RSV immuno-prophylaxis** ***(Options: Y/N)***
Considering a child, <u>within six months of the start of the RSV season</u>:				
Has received medical therapy (ie, supplemental oxygen, bronchodilator, diuretic, and/or corticosteroid)				
Has had resting tachypnea or respiratory distress				
Has had hypoxemia				
Has had feeding difficulties due to breathing problems				
Has had poor growth or weight gain due to breathing problems				
Has had gastroesophageal reflux and breathing difficulties				

5. Would you <u>recommend</u> RSV immunoprophylaxis for children with CLDP whom you identify as being at high risk for severe RSV disease <u>even if you suspect there would be poor parental compliance with the recommendation</u>? (*Options: Yes, I would recommend*

it despite my suspicion of poor compliance; No, I would not recommend it because of anticipated poor compliance).

6. If you do not recommend RSV immunoprophylaxis for all high-risk children with CLDP, what are the top <u>three most common reasons</u> for a lack of recommendation? *(Options include the following: allow for selection of up to three answers)*
   a. *Contraindication or allergy to RSV immunoprophylaxis*
   b. *Low platelet count*
   c. *Burden of monthly injections*
   d. *Perceived parental noncompliance*
   e. *Parental refusal*
   f. *Lack of or insufficient insurance*
   g. *Perceived financial burden to the family*
   h. *Lack of demonstrated cost-effectiveness of RSV immunoprophylaxis*
   i. *Concerns regarding efficacy of RSV immunoprophylaxis*
   j. *Concerns regarding safety of RSV immunoprophylaxis*
   k. *Other (please specify)*

7. Of all children with CLDP to whom you recommend RSV immunoprophylaxis, approximately what percentage of children in your practice receive all monthly dose throughout the RSV season? *(Open-ended)*

8. If a child with CLDP began RSV immunoprophylaxis at the beginning of the RSV season, but turned 24 months old during the RSV season, do you recommend stopping the prophylaxis, or do you continue dosing throughout the RSV season? *(Options: Stop; Continue; Not applicable) (If "Stop," then continue to question 8a, otherwise, proceed to question 9)*
   a. What would be the reasons for stopping therapy? *(Options [select as many as apply]: parental request, insurance company coverage, provider recommendation; other [please specify])*

9. A child with CLDP who is eligible for RSV immunoprophylaxis arrives at your office or clinic and the RSV season has already begun. How many doses would you recommend that this child receive? (*Options: starting dose followed by once a month until end of season; no doses; other [please specify])*

10. Do you ever recommend or prescribe more than 5 doses of RSV immunoprophylaxis during a single RSV season to a child with CLDP? *(Options: yes; no) (If "yes," proceed to question 10a, otherwise, proceed to question 11)*

a. under what circumstances? *(open-ended)*

11. Of the children with CLDP to whom you recommend RSV immunoprophylaxis in their first RSV season, do you ever recommend that they receive it in their second RSV season? *(Options: yes, no) (If "no," proceed to question 11a then question 12; if "yes," proceed to question 11b, then 11c, and then 12)*
    a. please explain why. (open-ended)
    b. Under what circumstances?
    c. approximately what percentage of those who receive RSV immunoprophylaxis in their first RSV season continue to receive it in their second RSV season? *(open-ended)*

Section 3: Administration of RSV Immunoprophylaxis

12. Does the primary hospital with whom you're affiliated provide the first dose of RSV immunoprophylaxis to your patients prior to the birth discharge?
    a. Yes, the primary hospital with which I am affiliated provides the first dose of RSV immunoprophylaxis prior to discharge. (*go to question 13, then 14)*
    b. No, the primary hospital with which I am affiliated does not provide the first dose of RSV immunoprophylaxis prior to discharge. (*go to question 15)*
    c. I am not affiliated with any specific hospital (*go to question 15)*

13. If the primary hospital with which you are affiliated provides the first dose of RSV immunoprophylaxis prior to discharge, how are the remaining doses administered? *Responses should add up to 100%.*
    a. All subsequent doses administered at your office or clinic *(Numeric)*
    b. The next dose administered at your office or clinic and the remainder through the primary care provider/pediatrician's office *(Numeric)*
    c. The next dose administered at an outpatient facility (either primary care or pulmonology); with the outpatient facility making arrangements for subsequent doses to be administered by a home health agency *(Numeric)*
    d. All subsequent doses administered through the primary care provider/pediatrician. *(Numeric)*
    e. All subsequent doses administered at home by home health agency *(Numeric)*
    f. Other *(please specify)*

14. For hospital-administered doses, which specialist is primarily responsible for prescribing RSV immunoprophylaxis to your patients? *(Options (rank the responses in order of frequency if more than one response option applies): Pediatric Pulmonologist; Pulmonology intensivist; Neonatologist; Infectious disease specialist; Pediatrician; don't know)*

15. If the primary hospital with which you are affiliated does not provide the first dose of RSV immunoprophylaxis to your patients prior to discharge, or if you are not affiliated with a hospital, how do children for whom you recommend RSV immunoprophylaxis receive it? *Responses should add up to 100%.*
    a. All doses administered at your office or clinic *(Numeric)*
    b. First dose administered at your office or clinic; subsequent doses administered through the primary care provider/pediatrician. *(Numeric)*
    c. First dose administered at an outpatient facility (either primary care or pulmonology); with the outpatient facility making arrangements for subsequent doses to be administered by a home health agency *(Numeric)*
    d. All doses administered through the primary care provider/pediatrician. *(Numeric)*
    e. All doses administered at home by home health agency *(Numeric)*

16. *(This question will only appear if 13b, 13c, 13d, 13e, 15b, 15c, 15d, and/or 15e are selected)* If RSV immunoprophylaxis doses are to be administered to your patients by the primary care provider/pediatrician or through a home health agency, how is this information communicated to the primary care provider/pediatrician? *(Options include the following: select as many as apply)*
    a. *Complete a prescription and give it to the parent(s)*
    b. *Fax/letter to primary care provider/pediatrician*
    c. *Telephone or email (ie, directly contact) the primary care provider/pediatrician*
    d. *By unified electronic medical record*
    e. *Combination of the above*
    f. Other *(please specify)*

17. *(This question will only appear if 13a, 15a, and/or 15b are selected)* For doses that are administered at your office or clinic, how do you assure that the child comes back for follow-up visits to receive their RSV immunoprophylaxis injection? *(Options [select as many as apply]):*
    a. Electronic reminders for parents (eg, email)
    b. Written reminders for parents by mail
    c. Reminder for yourself (eg, tracking sheet)
    d. Electronic health record pop-out
    e. Schedule the next RSV immunoprophylaxis injection at the end of the appointment for the current injection
    f. Other *(please specify)*

18. *(This question will only appear if 13a, 15a and/or 15b are selected)* For doses that are administered at your office or clinic, if a child misses an appointment to receive a RSV immunoprophylaxis injection, do you notify their parent(s) of their missed appointment?

(Options: yes; no) (If "yes," then proceed to question 18a and then 18b, else go to question 19)

a. How do you notify them? *(select as many as apply)*
   i. *Telephone*
   ii. *Postcard/letter*
   iii. *Electronic reminder*
   iv. *Other (please specify)*
b. Would you also inform the child's primary care provider of the missed dose? *(Options: yes; no)*

19. *(This question will only appear if 13a, 15a, and/or 15b NOT are selected)* If the RSV immunoprophylaxis doses are not administered at your office or clinic, how do you assure that the child comes back for follow-up visits with you to check on their medical status? *(Options include the following: select as many as apply)*
    a. Electronic reminders for parents (eg, email)
    b. Written reminders for parents, by mail
    c. Reminder for yourself (eg, tracking sheet)
    d. Electronic health record pop-up
    e. Schedule a follow-up visit at the end of the current visit
    f. Other *(please specify)*

20. If a child misses a regular follow-up visit with you regarding their medical status, do you notify their parent(s) of their missed appointment? *(Options: yes; no) (If "yes," then proceed to question 20a, otherwise, proceed to question 21)*
    a. How do you notify them? *(select as many as apply)*
       i. *Telephone*
       ii. *Postcard/letter*
       iii. *Electronic reminder*
       iv. *Other (please specify)*

21. *(This question will only appear if 13a, 15a, and/or 15b NOT are selected)* If the RSV immunoprophylaxis doses are not administered at your office or clinic, how are you kept informed of the status of the administration of the RSV immunoprophylaxis doses, occurring through the primary care provider/pediatrician/home health agency? *(Select as many as apply)*
    a. Electronic letter/notification
    b. Written letter/notification
    c. Unified health record
    d. Immunization card
    e. Parental report
    f. Other (please specify)

Section 4: Access to RSV Immunoprophylaxis

22. Are there any patient groups for whom you think RSV immunoprophylaxis should be provided but is not currently recommended by the 2012 AAP guidelines? *(Options: yes; no) (If "yes," then proceed to question 22a, otherwise proceed to question 23)*
    a. which groups? *(Options (select as many as apply): those with cystic fibrosis; those with congenital anomalies of the respiratory system; those with congenital diaphragmatic hernia; those with history of mechanical ventilation; those with history of meconium aspiration; other [please specify])*

23. What is the biggest obstacle in getting RSV immunoprophylaxis to your patients? *(Options [rank top three]: you forget; insurance denials; limitations to commercial insurance coverage; limitations to Medicaid coverage; unclear eligibility criteria; noncompliance; communication gaps between hospital discharge team and prescriber; communication gaps between primary care provider and pediatric pulmonologist; child's family stability; reliability of child's parents/caretakers; other [please specify])*

24. What would be the biggest help to you to facilitate provision of RSV immunoprophylaxis to your patients? *(Options (rank top three): more training or seminars provided to neonatologists and primary care providers specific to pulmonary risk factors; more training or seminars provided to pediatric pulmonologists; written "cheat sheets" reminding you of the eligibility criteria; electronic health record pop-up reminders; detail sales representatives; quality improvement modules specific for RSV immunoprophylaxis; insurance letter templates; cost-effectiveness data; other [please specify])*

25. In your opinion, what is the best tool you could have to ensure that eligible children get RSV immunoprophylaxis? *(Options [rank top three]): Electronic health record pop-up reminders; pulmonology involvement in the discharge planning process; practice-based reminder schedule on paper or computer; reminder at each child encounter by sticker or electronic; your own memory; parental education prior to discharge; other [please specify])*

26. Would you like to receive honoraria for completing the survey? *(Options: yes, no) (If no, proceed to question a, if 'yes' proceed to question b)*
    a. Thank you very much for participating in the survey.
    b. Which of the following do you prefer? *(only allow participant to select one of the following)*
        i. A $75 Amazon voucher *(Options: yes, no) (If yes, ask for the following)*
            1. Your name*(open-ended)*
            2. Your work address *(open-ended)*

3. Your email address *(open-ended)*

ii. Donation of $75 to UNESCO *(Options: yes, no) (If "yes," guide them for donation*

Appendix III

Screener and Survey for Pediatric Cardiologists

Screening

1. Please indicate:
    a. The geographic location of your practice *(Options: drop-down menu with state names)(proceed to next question)*
    b. Your sex (*Options: male; female) (proceed to next question)*
    c. Your specialty *(Options: Pediatric Cardiology; Adult Cardiology; None of the above) (if 'Adult Cardiology' or 'None of the above', screener ends, else proceed to next question)*
    d. Considering the sample of children in your practice with characteristics that put them at high risk for severe respiratory syncytial virus (RSV) disease, do you recommend RSV immunoprophylaxis? *(Options: yes; no) (if "no," proceed to question 1di, after which the participant is not eligible to continue with survey, if "yes," proceed to 1dii and then proceed to survey)*
        i. why? *(open-ended)*
        ii. please provide the approximate number of patients for whom you have recommended RSV immunoprophylaxis in the past 12 months? *(open-ended)*

Section 1: Respondent/Practice Information

1. Please indicate your:
    a. Year of graduation from fellowship *(open-ended)*
    b. Primary practice type (*Options: medical school-based teaching hospital; non-medical school teaching hospital; non–teaching community hospital; large group practice (≥5 pediatric specialists); small group (<5 pediatric specialists); or individual practice)*
    c. Primary area of pediatric cardiology (*Options (select as many as apply): Outpatient; Catheterization lab; Imaging; Intensive care unit; Inpatient ward; Research)*

2. What type of community does your primary practice serve? *(Options, select as many options as apply]: Inner city; Urban [non–inner city]); Suburban; Rural)*

3. Please indicate the approximate number of children (24 months of age or younger) within your practice with hemodynamically significant congenital heart disease (HS-CHD):
    a. Whom you cared for in the past 12 months *(Open-ended)*

b. The proportion of those whom you cared for in the past 12 months, who received RSV immunoprophylaxis in the past 12 months *(Options: <20%; 20%–39%; 40%–59%; 60%–79%; ≥80%)*

4. Are pediatric cardiac surgeries performed at the hospital with which you are primarily affiliated? *(Options: yes; no) (If "yes," then proceed to question 4 a, else proceed to question 5)*
   a. approximately how many pediatric cardiac surgeries are conducted per year? *(Options: <250; 250–499; ≥500, don't know)*

Section 2: Current Practice

5. The 2012 American Academy of Pediatrics (AAP) guidelines for RSV immunoprophylaxis state: "Children who are 24 months of age or younger with hemodynamically significant cyanotic or acyanotic CHD may benefit from palivizumab prophylaxis. Decisions regarding prophylaxis with palivizumab in children with CHD should be made on the basis of the degree of physiologic cardiovascular compromise. Children younger than 24 months of age with CHD who are most likely to benefit from immunoprophylaxis include:
   - Infants who are receiving medication to control congestive heart failure
   - Infants with moderate to severe pulmonary hypertension
   - Infants with cyanotic heart disease

   Because a mean decrease in palivizumab serum concentration of 58% was observed after surgical procedures that use cardiopulmonary bypass, for children who still require prophylaxis, a postoperative dose of palivizumab (15 mg/kg) should be considered as soon as the patient is medically stable."

   For each factor considered below, please provide the approximate percentage of children with HS-CHD (24 months of age or younger at start of RSV season) that you would expect to have the specified condition, in your practice. Please also identify if the following factors are indicative of: a) HS-CHD; b) high risk for severe RSV disease, and/or c) eligibility for RSV immunoprophylaxis?

	**Expected prevalence (%), among children with HS-CHD in your practice** ***(open-ended)***	**Indicative, in whole or in part, of HS-CHD** ***(Options: Y/N)***	**At high risk for severe RSV disease** ***(Options: Y/N)***	**Eligible for RSV immuno-prophylaxis** ***(Options: Y/N)***
Who are receiving medication to control congestive heart failure				
With moderate to severe pulmonary hypertension				
With cyanotic heart disease				
With prior severe respiratory illness				
With recent (within 6 weeks) cardiac surgery				
Who had full/complete corrective cardiac surgery, 6–12 *weeks* previously				
Who had full/complete corrective cardiac surgery, >3 months previously				
Who had partial corrective surgery/palliative intervention, 6–12 weeks previously				
Who had partial corrective surgery/palliative intervention >3 months previously				
With cardiac surgery planned during RSV season				
With chromosomal abnormalities (eg, Down syndrome, diGeorge syndrome)				
With other comorbid pulmonary conditions				

6. For patients receiving RSV immunoprophylaxis who undergo surgery with cardiopulmonary bypass:
    a. Would you recommend a postoperative dose of RSV immunoprophylaxis as soon as the patient is medically stable? *(Options: yes; no) (If "no," proceed to question 6ai then proceed to question 7, else go to question 6b)*
        i. when would you recommend the next dose to be administered, and with what frequency? *(open-ended)*
    b. If you would recommend a postoperative dose of RSV immunoprophylaxis as soon as the patient is medically stable, would you then recommend restarting monthly doses if the season has not yet ended? *(Options: yes; no) (If "yes," then proceed to question 6bi, else go to question 7)*
        i. when? *(Options: monthly from postoperative dose; monthly from discharge; other (please specify))*

7. Would you <u>recommend</u> RSV immunoprophylaxis for children with HS-CHD who you identify as being at high risk for severe RSV disease <u>even if you suspect there would be poor parental compliance with the recommendation</u>? (*Options: Yes, I would recommend it despite my suspicion of poor compliance; No, I would not recommend it because of anticipated poor compliance).*

8. If you do not recommend RSV immunoprophylaxis for all high-risk children with HS-CHD, what are the top <u>three most common reasons</u> for a lack of recommendation? *(Options include the following: allow for selection of up to three answers)*
    a. Contraindication or allergy to RSV immunoprophylaxis
    b. Low platelet count
    c. Burden of monthly injections
    d. Perceived parental noncompliance
    e. Parental refusal
    f. Lack of or insufficient insurance
    g. Perceived financial burden to the family
    h. Lack of demonstrated cost-effectiveness of RSV immunoprophylaxis
    i. Concerns regarding efficacy of RSV immunoprophylaxis
    j. Concerns regarding safety of RSV immunoprophylaxis
    k. Other (please specify)

9. Of all children with HS-CHD to whom you recommend RSV immunoprophylaxis, approximately what percentage of children in your practice receive all monthly dose throughout the RSV season? *(open-ended)*

10. If a child with HS-CHD began RSV immunoprophylaxis at the beginning of the RSV season, but turned 24 months old during the RSV season, do you recommend stopping

the RSV immunoprophylaxis, or do you continue dosing throughout the RSV season? *(Options: Stop; Continue; Not applicable) (If 'Stop' then continue to question 10a, otherwise, proceed to question 11)*

   a. What would be the reasons for stopping therapy? *(Options (select as many as apply): parental request; insurance company coverage; provider recommendation; Other (please specify))*

11. A child with HS-CHD who is eligible for RSV immunoprophylaxis arrives at your office or clinic and the RSV season has already begun. How many doses would you recommend that this child receive? (*Options: starting dose followed by once a month until end of season; no doses; other (please specify))*

12. Do you ever recommend or prescribe more than 5 doses of RSV immunoprophylaxis during a single RSV season to a child with HS-CHD? *(Options: yes; no) (If 'yes', proceed to question 12a, otherwise, proceed to question 13)*
    a. under what circumstances? *(open-ended)*

13. Of the children with HS-CHD to whom you recommend RSV immunoprophylaxis in their first RSV season, do you ever recommend that they receive it in their second RSV season? *(Options: yes, no) (If 'no' proceed to question 13a then question 14, if 'yes' proceed to question 13b, then 13c, then question 14)*
    a. please explain why. *(open-ended)*
    b. Under what circumstances?
    c. approximately what percentage of those who receive RSV immunoprophylaxis in their first RSV season continue to receive it in their second RSV season? *(open-ended)*

Section 3: Administration of RSV Immunoprophylaxis

14. Does the primary hospital with whom you're affiliated provide the first dose of RSV immunoprophylaxis to your patients prior to the birth discharge?
    a. Yes, the primary hospital with which I am affiliated provides the first dose of RSV immunoprophylaxis prior to discharge. (*go to question 15, then 16)*
    b. No, the primary hospital with which I am affiliated does not provide the first dose of RSV immunoprophylaxis prior to discharge. (*go to question 17)*
    c. I am not affiliated with any specific hospital (*go to question 17)*

15. If the primary hospital with which you are affiliated provides the first dose of RSV immunoprophylaxis to your patients prior to discharge, how are the remaining doses administered? *Responses should add up to 100%.*

a. All subsequent doses administered at your office or clinic *(Numeric)*
b. The next dose administered at your office or clinic and the remainder through the primary care provider/pediatrician's office *(Numeric)*
c. The next dose administered at an outpatient facility (either primary care or cardiology); with the outpatient facility making arrangements for subsequent doses to be administered by a home health agency *(Numeric)*
d. All subsequent doses administered through the primary care provider/pediatrician *(Numeric)*
e. All subsequent doses administered at home by home health agency *(Numeric)*
f. Other *(please specify)*

16. For hospital-administered doses, which specialist is primarily responsible for prescribing RSV immunoprophylaxis to your patients? *(Options [rank the responses in order of frequency if more than one response option applies]: Pediatric Cardiologist; Cardiac intensivist; Neonatologist; Pulmonologist; Infectious disease specialist; Pediatrician; don't know)*

17. If the primary hospital with which you are affiliated does not provide the first dose of RSV immunoprophylaxis to your patients prior to discharge, or if you are not affiliated with a hospital, how do children for whom you recommend RSV immunoprophylaxis receive it? *Responses should add up to 100%.*
    a. All doses administered at your office or clinic *(Numeric)*
    b. First dose administered at your office or clinic; subsequent doses administered through the primary care provider/pediatrician. *(Numeric)*
    c. First dose administered at an outpatient facility (either primary care or cardiology); with the outpatient facility making arrangements for subsequent doses to be administered by a home health agency *(Numeric)*
    d. All doses administered through the primary care provider/pediatrician. *(Numeric)*
    e. All doses administered at home by home health agency *(Numeric)*

18. *(This question will only appear if 15b, 15c, 15d, 15e, 17b, 17c, 17d, and/or 17e are selected)* If RSV immunoprophylaxis doses are to be administered to your patients by the primary care provider/pediatrician or through a home health agency, how is this information communicated to the primary care provider/pediatrician? *(Options include the following: select as many as apply)*
    a. *Complete a prescription and give it to the parent(s)*
    b. *Fax/letter to* primary care provider/*pediatrician*
    c. *Telephone or email (ie, directly contact)* the primary care provider/pediatrician
    d. By unified electronic medical record
    e. *Combination of the above*
    f. Other *(please specify)*

19. *(This question will only appear if 15a, 17a, and/or 17b are selected)* For doses that are administered at your office or clinic, how do you assure that the child comes back for follow-up visits to receive their RSV immunoprophylaxis injection? *(Options include the following: select as many as apply)*
    a. Electronic reminders for parents (eg, email)
    b. Written reminders for parents by mai*l*
    c. Reminder for yourself (eg, tracking sheet)
    d. Electronic health record pop-out
    e. Schedule the next RSV immunoprophylaxis injection at the end of the appointment for the current injection
    f. Other *(please specify)*

20. *(This question will only appear if 15a, 17a and/or 17b are selected*) For doses that are administered at your office or clinic, if a child misses an appointment to receive a RSV immunoprophylaxis injection, do you notify their parent(s) of their missed appointment? *(Options: yes; no) (If "yes,", then proceed to question 20a and then 20b, else go to question 21)*
    a. How do you notify them? *(select as many as apply)*
        v. Telephone
        vi. Postcard/letter
        vii. Electronic reminder
        viii. Other (please specify)
    d. Would you also inform the child's primary care provider of the missed dose? *(Options: yes; no)*

21. *(This question will only appear if 15a, 17a and/or 17b are NOT selected)* If the RSV immunoprophylaxis doses are not administered at your office or clinic, how do you assure that the child comes back for follow-up visits with you to check on their medical status? *(Options include the following: select as many as apply)*
    a. Electronic reminders for parents (eg, email)
    b. Written reminders for parents, by mail
    c. Reminder for yourself (eg, tracking sheet)
    d. Electronic health record pop-up
    e. Schedule a follow-up visit at the end of the current visit
    f. Other *(please specify)*

22. If a child misses a regular follow-up visit with you regarding their medical status, do you notify their parent(s) of their missed appointment? *(Options: yes; no) (If 'yes', then proceed to question 22a, otherwise, proceed to question 23)*
    a. How do you notify them? *(select as many as apply)*
        v. Telephone

    vi. *Postcard/letter*
    vii. *Electronic reminder*
    viii. *Other (please specify)*

23. *(This question will only appear if 15a, 17a, and/or 17b are NOT selected)* If the RSV immunoprophylaxis doses are not administered at your office or clinic, how are you kept informed of the status of the administration of the RSV immunoprophylaxis doses, occurring through the primary care provider/pediatrician/home health agency? *(Select as many as apply)*
    a. *Electronic letter/notification*
    b. *Written letter/notification*
    c. *Unified health record*
    d. *Immunization card*
    e. *Parental report*
    f. *Other (please specify)*

Section 4: Access to RSV Immunoprophylaxis

24. Are there any patient groups for whom you think RSV immunoprophylaxis should be provided but is not currently recommended by the 2012 AAP guidelines? *(Options: yes; no) (If "yes," then proceed to question 24a, otherwise proceed to question 25)*
    a. which groups? *(Options[select as many as apply]: those with early congestive heart failure; those expected to have surgery during the RSV season; those anticipated to develop HS-CHD; other [please specify])*

25. What is the biggest obstacle in getting *RSV immunoprophylaxis* to your patients? *(Options (rank top three): you forget; insurance denials; limitations to commercial insurance coverage; limitations to Medicaid coverage; unclear eligibility criteria; noncompliance; communication gaps between hospital discharge team and prescriber; communication gaps between primary care provider and pediatric cardiologist; child's family stability; reliability of child's parents/caretakers; other [please specify])*

26. What would be the biggest help to you to facilitate provision of *RSV immunoprophylaxis* to your patients? *(Options (rank top three): more training or seminars provided to neonatologists and primary care providers specific to cardiology risk factors; more training or seminars provided to pediatric cardiologists; written "cheat sheets" reminding you of the eligibility criteria; electronic health record pop-up reminders; detail sales representatives; quality improvement modules specific for RSV*

immunoprophylaxis; insurance letter templates; cost-effectiveness data; other [please specify])

27. In your opinion, what is the best tool you could have to ensure that eligible children get RSV immunoprophylaxis? *(Options (rank top three): Electronic health record pop-up reminders; cardiology involvement in the discharge planning process; practice-based reminder schedule on paper or computer; reminder at each child encounter by sticker or electronic; your own memory; parental education prior to discharge; other (please specify))*

28. Would you like to receive honoraria for completing the survey? *(Options: yes, no) (If "no," proceed to question a, if "yes," proceed to question b)*

    a. Thank you very much for participating in the survey.
    b. Which of the following do you prefer? *(only allow participant to select one of the following)*
        i. A $75 Amazon voucher *(Options: yes, no) (If yes, ask for the following)*
            4. Your name*(open-ended)*
            5. Your work address *(open-ended)*
            6. Your email address *(open-ended)*
        ii. Donation of $75 to UNESCO *(Options: yes, no) (If "yes," guide them for donation)*

Appendix IV.
Schematic of the Number of Invited and Eligible Respondents Who Started and Completed the Survey

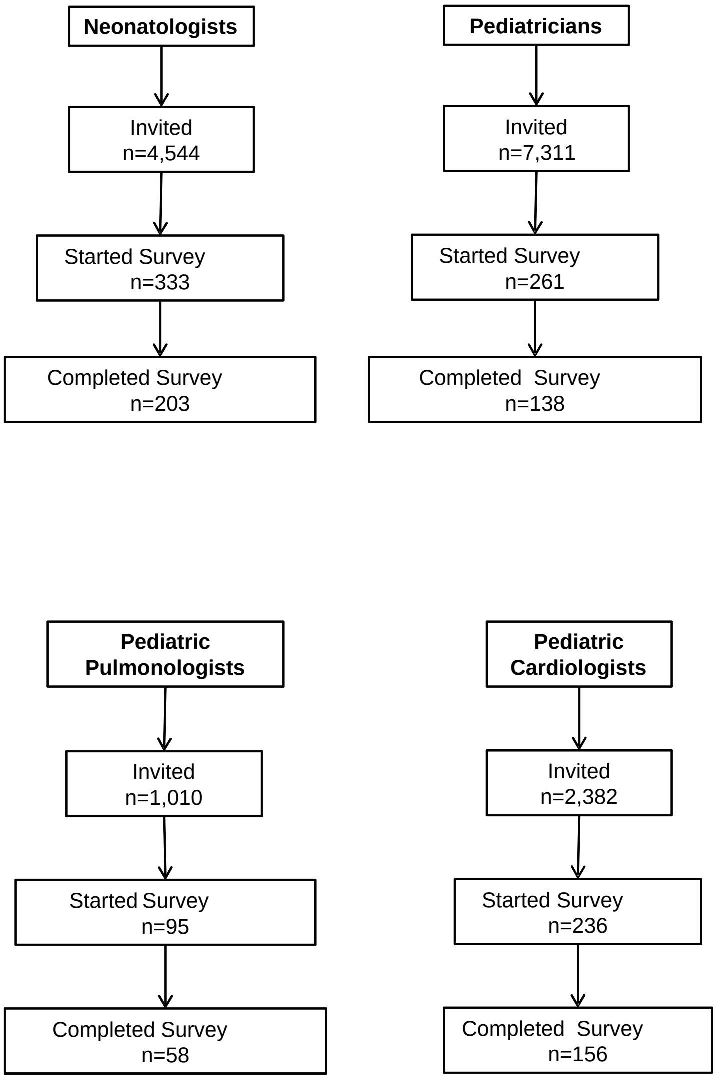

Supplement: Supplementary material [file CLP621343.pdf]
